# Identification of intra-abdominal lymphatics in canine carcasses by laparoscopic fluorescence lymphography with intradermal and intrapopliteal ICG administration

**DOI:** 10.1371/journal.pone.0241992

**Published:** 2020-11-12

**Authors:** Francisco M. Sánchez-Margallo, Maurício Veloso Brun, Juan A. Sánchez-Margallo

**Affiliations:** 1 Scientific Direction, Jesús Usón Minimally Invasive Surgery Centre, Cáceres, Spain; 2 Department of Small Animal Clinics (DCPA), Santa Maria, Universidade Federal de Santa Maria (UFSM), Programa de Pós-graduação em Medicina Veterinária (PPGMV) of UFSM Researcher of Conselho Nacional de Desenvolvimento Científico e Tecnológico (CNPq), Camobi, RS, Brazil; 3 Bioengineering and Health Technologies Unit, Jesús Usón Minimally Invasive Surgery Centre, Cáceres, Spain; Louisiana State University, UNITED STATES

## Abstract

**Objective:**

To evaluate the feasibility of laparoscopic fluorescence lymphography (LFL) using indocyanine green (ICG) via intradermal (ID) or intrapopliteal (IPP) administration in an ex vivo canine model.

**Methods:**

Six thawed adult male dog carcasses were placed in the Trendelenburg position after placing three surgical ports in triangulation. ICG (0.5 mg/ml; 0.05 mg/kg) was administered to five of the carcasses (one carcass was a pilot) via ID in the left torso and IPP (right position, by surgical access) to stain the lymphatic pathway and medial iliac lymph node (MILN). Fluorescence quality, staining time, structures stained, and lymph node histopathology were assessed. Thoracic duct staining was also evaluated.

**Results:**

ID administration showed staining of parts of the lymphatic pathway of the iliosacral lymph center in all cases using a single dose of ICG, with left MILN visualization in four carcasses. IPP administration showed staining of the right MILN in all cases, using a single dose in four carcasses. ICG reached the thoracic duct in one case. The two administration routes showed similar results in terms of required ICG volume, staining time, and visualization quality, although IPP was more effective in staining the MILN.

**Conclusions:**

This study confirms the feasibility of staining the iliosacral lymph center (mainly the MILNs) by LFL in thawed dog carcasses via ID or IPP administration of ICG. However, the IPP route showed greater effectiveness in staining the MILN.

## Introduction

Surgery of the lymphatic ducts and lymph nodes in dogs is of increasing importance because of its relevance to the staging, prognosis, and treatment of malignancies [1, 2] as well as its application to the demarcation of the thoracic duct and its branches for treatment of chylothorax [3]. In addition, given the established role of lymphadenectomy in oncological diseases involving different medical-surgical specialties, further studies on lymphatic pathways and lymph node staining in dogs are needed to improve the development and outcomes of this procedure.

The lymphatic dyes used in dogs included octafluoropropane-filled lipid microspheres [4], patent blue V [5], patent blue *plus* technetium [6], methylene blue [7, 8], isosulfan blue [9], hemosiderin plus technetium [6], iopamidol [3, 10], indocyanine green (ICG) [3], and ICG plus methylene blue [3]. The use of ICG as a direct near-infrared fluorescent marker in video surgery is still under investigation in dogs. However, it has shown applicability in the intraoperative identification of hepatocellular carcinoma under laparotomy [11], analysis of blood supply in anorectal autotransplantation [12], vessel identification and graft patency in robot-assisted endoscopic coronary artery bypass [13], lymphatic duct identification during thoracoscopic thoracic duct ligation [3], detection of sentinel lymph nodes in the oral mucosa [14], and for lymphography and lymphangiography in vascularized lymph node transfer [9].

ICG is frequently administered intravenously [11–13], via the popliteal lymph node (with or without surgical dissection) and the mesenteric lymph nodes [3], subcutaneously (SC) around the anus [15], into the buccal submucosa [14], and intradermally [9, 16].

A previous study in ex vivo canine models showed that intradermal (ID) administration of ICG allowed the identification of the superficial lymphatic system in thawed dog carcasses [17]. Another study demonstrated that intrauterine administration of patent blue via an open approach allowed staining of the medial iliac lymph nodes (MILNs) in fresh dog carcasses [5]. Based on the reported effectiveness of ICG staining in ex vivo canine models, we hypothesized that the use of ICG in laparoscopic near-infrared fluorescent lymphography might also be a feasible way to stain the abdominal and/or pelvic lymphatic ducts and MILNs in thawed dog carcasses. Confirmation of this hypothesis may provide new possibilities for the further development of anatomical and surgical studies in ex vivo canine models involving fluorescent lymphography.

The aim of this study was to evaluate the feasibility of laparoscopic fluorescence lymphography (LFL) using ICG via ID or intrapopliteal (IPP) administration in an ex vivo canine model. To our knowledge, this is the first study to investigate this possibility, demonstrating the efficacy of staining the nodes of the iliosacral lymphatic center through ICG administration via IPP or ID in the torso of thawed dog carcasses.

## Material and methods

The present study used six thawed male Beagle dog carcasses euthanized for purposes other than this research. The animals used in this study were reused from previous training or research studies that were approved by the Ethical Committee and Institutional Animal Care of the Minimally Invasive Surgery Centre (MISCJU). The first carcass (C1) was used as a pilot study. The ventral abdominal region, left torso, right thorax, and right popliteal lymph node region was shaved in all animals.

**Fig 1:**
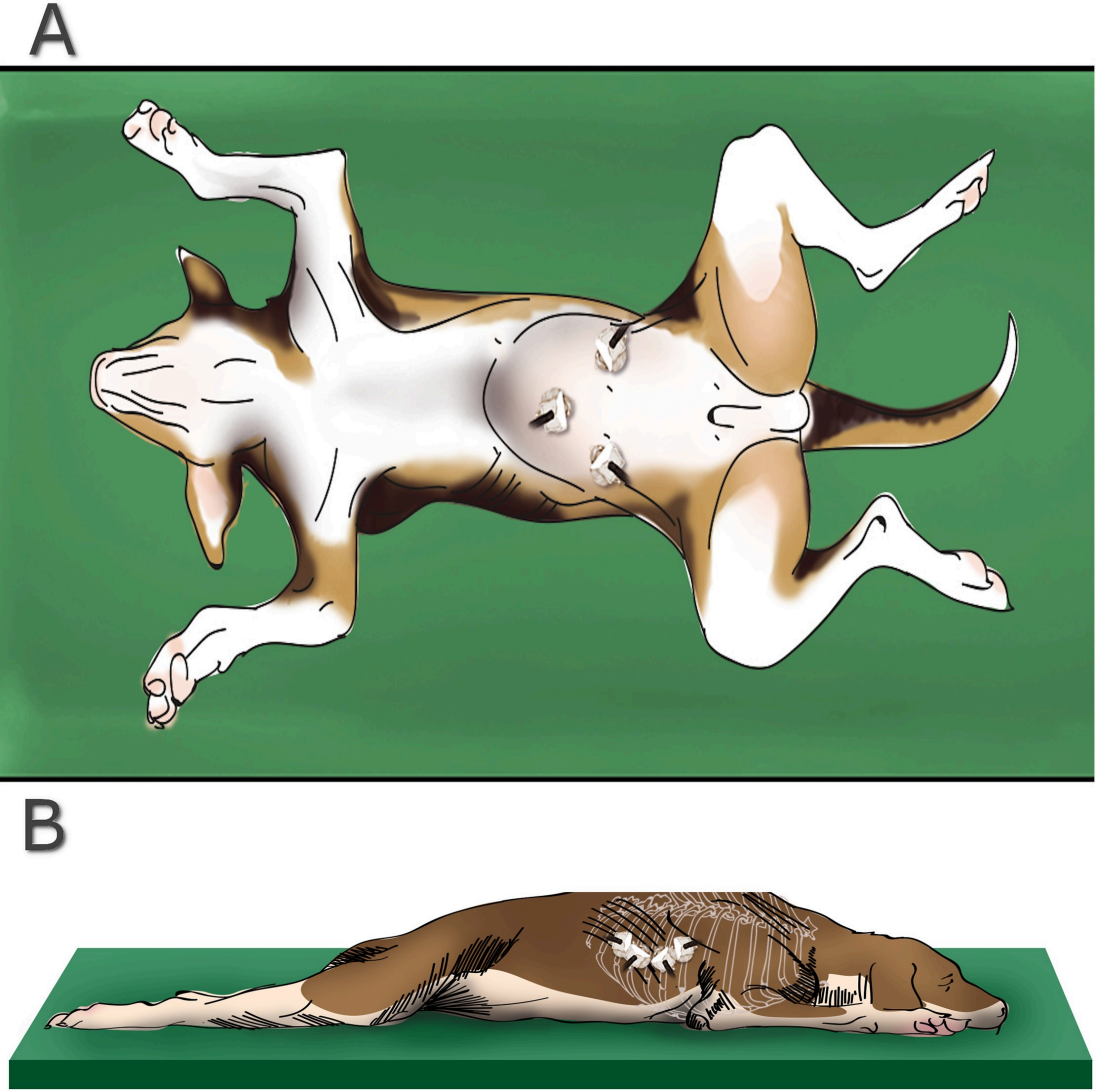
Location of the trocars for access to the (a) pelvic and (b) thoracic cavity.

**Fig 2:**
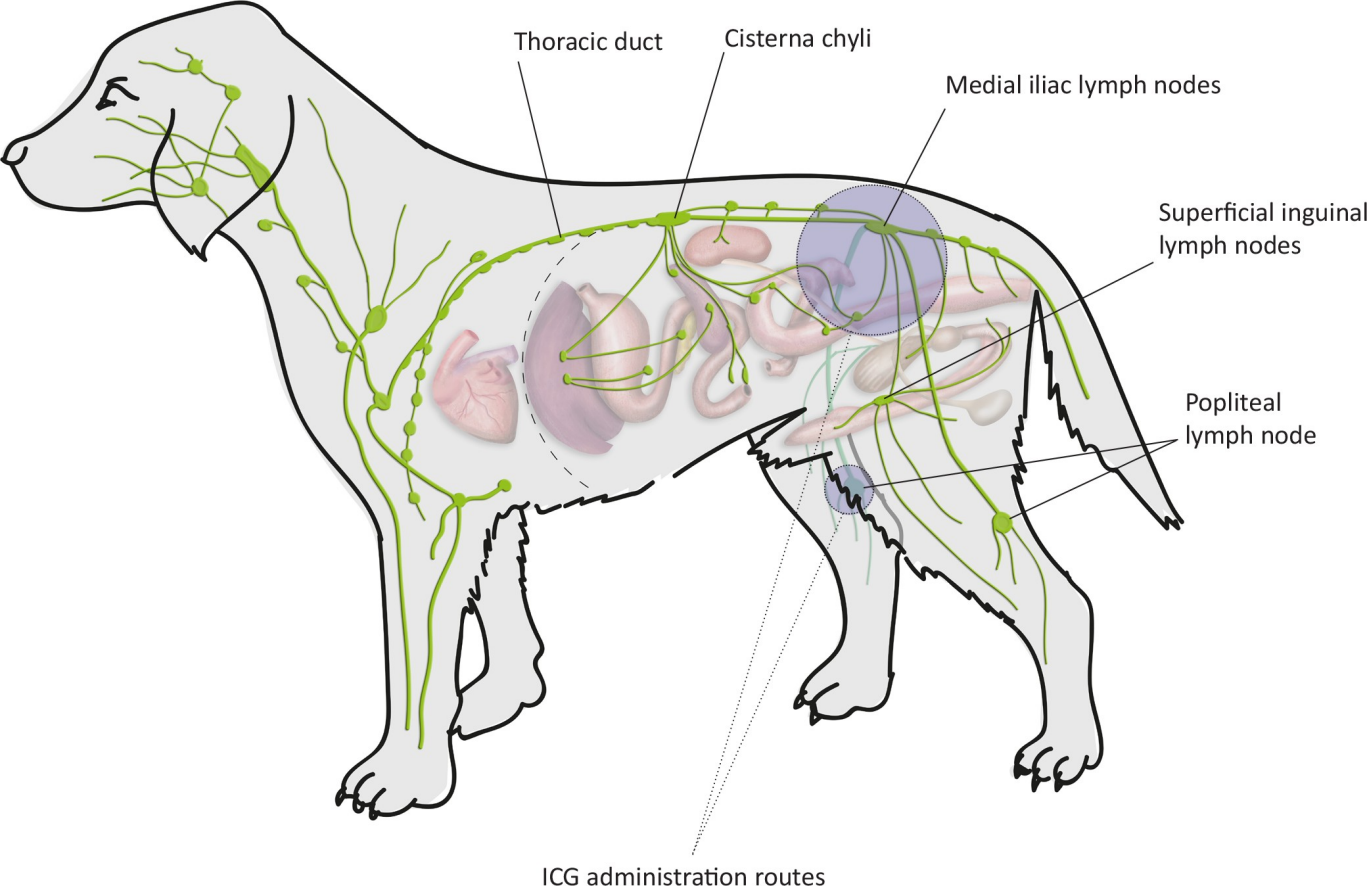
Canine lymphatic anatomy analysed and areas of ICG administration.

With each animal in the supine position, a cutaneous incision in the umbilical scar was made to insufflate the peritoneal cavity with medicinal CO_2_ (12 mmHg at 5 Lmin) using a modified Hasson technique. An 11-mm trocar (Versaport; Covidien, Dublin, Ireland) was placed through this surgical access port and two other cannulas, 11 mm and 5 mm (Versaport; Covidien), were placed in triangulation with the first trocar (Fig 1A). Each carcass was then placed in a Trendelenburg position with the abdomen rotated to the right, exposing the left flank for intradermal ICG administration in the torso. The peritoneal cavity was irrigated with 0.9% NaCl. Subsequently, the lavage fluid and residual blood were aspirated. In each dog, 0.05 mg/kg of ICG (Verdye, 5 mg/ml, Diagnostic green GmbH, Aschheim, Germany), was diluted in 0.9% NaCl until 0.5 mg/ml and it was administered via ID at different points in the left torso region (Fig 2). The torso was massaged continuously while the intraabdominal area corresponding to the lymphatic pathway and MILN was assessed by laparoscopy. A 10-mm and 0-degree optic coupled to a video system (Image1 Spies TC 200; Karl Storz SE & Co. KG, Tuttlingen, Germany) and a light source (SCB D-light P; Karl Storz SE & Co. KG) were used to evaluate luminescence. The xenon light source provides both visible and NIR excitation light at a wavelength of 690 nm to 790 nm. All procedures were recorded for further evaluation of staining times and transoperative findings.

For the IPP administration of ICG, the right popliteus of all animals was accessed by a cutaneous incision and blunt dissection to expose the lymph node with minimal dissection of adjacent adipose tissue to reduce interference in the lymphatic pathway (Fig 2). The same dose of ICG was administered as by the ID route. The administration was slow and carried out in pulses, as described previously [3]. Continuous massage, flexion, and extension of the right pelvic limb were performed.

Another ICG dose was administered every 15 min and up to a maximum of four additional doses were administered in both routes in the absence of fluorescence. During the entire evaluation period (up to 15 min after the last dose), the presence or absence of the lymphatic pathway and MILN staining were considered as well as the occurrence of other abdominal or pelvic lymph/micro lymph nodes, considering previous anatomical description [18]. The degree of staining of each structure was rated from 0 to 3 [3] (Table 1) and the range of staining time from I to V (Table 2). When more than one MILN was stained, only the staining time of the first was considered. Six carcasses were considered for the description of intraoperative findings. However, the five carcasses subjected to the same dose of ICG (C2-C6) were considered for the statistical analysis of total ICG volume, staining time, and staining quality.

**Table 1:** Quality of lymphatic pathway and lymph node visualizations in thawed dog carcasses [3].

Classification of the quality of the final visibility	Characteristics
0	Absent
1	Fluorescence/optical coloration present but fainter than optimal
2	Ideal fluorescence/optical coloration
3	More intense fluorescence/optical coloration than ideal

**Table 2:** Classifications of time intervals for laparoscopic fluorescence lymphography.

Classification	Time (min)
I	<3
II	≥3 and <10
III	≥10 and <20
IV	≥20 and <30
V	≥30 min

Fifteen minutes after the last ICG administration (right side), bilateral lymph nodes were collected using a laparoscopic approach and the samples were placed in separate vials for histological analysis. Subsequently, five of the six cadavers were placed in sternal recumbency for thoracoscopic evaluation of thoracic duct staining. In the pilot study (C1), thoracoscopy was performed before lymphadenectomy. To carry out the thoracoscopy, two 10.5 mm trocars (Thoracoport; Covidien) and a 5 mm trocar (Vesaport; Covidien) were placed between the 7th and 10th right intercostal spaces (Fig 1B). The mediastinal pleura were dissected to identify the fluorescence of the thoracic duct.

Wilcoxon signed-rank tests were used to compare ICG volumes, staining times, and quality of visualization between the administration routes (ID and IPP). All statistical analyses were performed using R version 3.6.1 (R Foundation for Statistical Computing, Vienna, Austria). The results are shown as means and standard deviation. For all tests, p < 0.05 was considered statistically significant.

## Results

C1 served as a pilot to verify the feasibility of ICG direct fluorescence by video surgery on a thawed dog carcass. The estimated weight of this animal was 25.0 kg and the ICG dose was >0.06 mg/kg. Once the bilateral lymphatic pathway and the MILN staining were verified in the pilot study, all remaining carcasses (C2–C6) were weighed after thawing and just before the surgeries. The average weight of these carcasses was 15.9±4.2 kg (16.5–22.0 kg). The transoperative results are summarized in Tables 3 and 4.

**Table 3:** Distributions of the structures visualized by LFL via intradermal administration of ICG in the torso of thawed dog carcasses (C1–C6) according to ICG dose (0.05%), staining time, and visualization quality.

Carcass (C#)	Intra-abdominal lymph vessels			MILN_L_			Other stained structures/observations
	*Dose (mg/kg)*	*Staining time*	*Visualization quality*	*Dose (mg/kg)*	*Staining time*	*Visualization quality*	
C1 (Pilot)	NC	NC	2	NC	NC	2	- Lymph vessel(s) between MILNs
							- 2 MILN_L_
C2	0.05	II	2	AS	AS	AS	- MILN_L_ not stained (dose: 2.00 mg/kg; time: III)
C3	0.05	II	2	AS	AS	AS	- MILN_L_ not stained (dose: 2.00 mg/kg; time: III)
C4	0.05	II	2	0.05	II	2	- Lymph vessel(s) between MILNs
C5	0.05	I	2	0.05	II	2	-
C6	0.05	I	2	0.10	IV	2	-

LFL, laparoscopic fluorescence lymphography; ICG, indocyanine green; MILN, medial iliac lymph node; MILN_L_, left MILN; MILN_R_, right MILN; IILN_R_, right internal iliac lymph node; AS, absence of staining; NC, not considered.

**Table 4:** Distributions of the structures visualized by LFL via intrapopliteal administration of ICG in thawed dog carcasses (C1–C6) according to ICG dose (0.05%), staining time, and visualization quality.

Carcass (C#)	Intra-abdominal lymph vessels			MILN_R_			Other structure staining/observations
	*Dose (mg/kg)*	*Staining time*	*Visualization quality*	*Dose (mg/kg)*	*Staining time*	*Visualization quality*	
C1 (Pilot)	NC	NC	2	NC	NC	2	-
C2^§^	0.05	I	2	0.05	I	2	- Communication between MILNs
							- Thoracic duct
C3	0.05	I	2	0.05	I	2	-
C4	0.05	II	2	0.05	III	2	- Communication between MILNs
C5*	0.10	IV	2	0.10	IV	2	- IILN_R_ (dose: 0.15 mg/kg; time: IV)
C6*	0.05	II	2	0.05	II	2	- Right abdominal and pelvic venous vessels (including external iliac and cava) (dose: 0.10 mg/kg; time: IV)
							- IILN_R_ not stained

LFL, laparoscopic fluorescence lymphography; ICG, indocyanine green; MILN, medial iliac lymph node; MILN_L_, left MILN; MILN_R_, right MILN; IILN_R_, right internal iliac lymph node; AS, absence of staining; NC, not considered.

^§^ Ureter section occurred during biopsy of the dyed tissue.

* Part of ICG dose administered around the lymph node.

**Fig 3:**
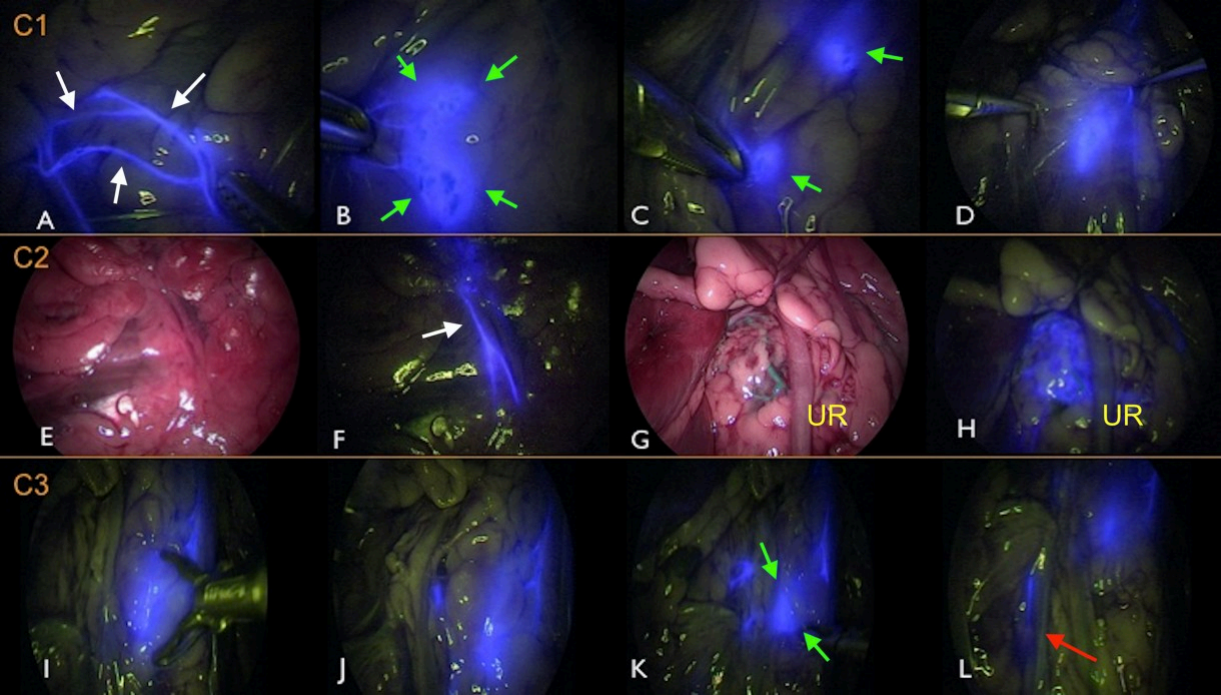
Laparoscopic fluorescence lymphography (LFL) in carcass 1 (C1) (A-D), C2 (E-H), and C3 (I-L). In A, B, C, E, and F, indocyanine green (ICG) was administered intradermally in the torso. In D, G, H, I, J, K, and L, ICG was administered via IPP. The blue stain shows the medial iliac lymph node (MILN) (white arrows) and/or efferent lymphatic pathways (green arrows). In C1, the left efferent lymph vessels (A) and the left MILN (B) are stained while entering the abdominal cavity along with the deep circumflex iliac vessels. Two left MILNs (C) and one right MILN (D) are shown. Efferent lymphatic pathways without (E) and with (F) near-infrared fluorescence filtering and right MILN without (G) and with (H) near-infrared fluorescence filtering are visible in C2. In C3, the right MILN (I, J, and L) and the lymph ducts (K, with green narrows) communicate with the left MILN (not stained). (L) shows the afferent lymphatic vessel (red arrow) to the cisterna chyli. In all carcasses, ICG fluorescence improved (or allowed) visualization of the MILN and lymphatic pathways. UR = ureter.

**Fig 4:**
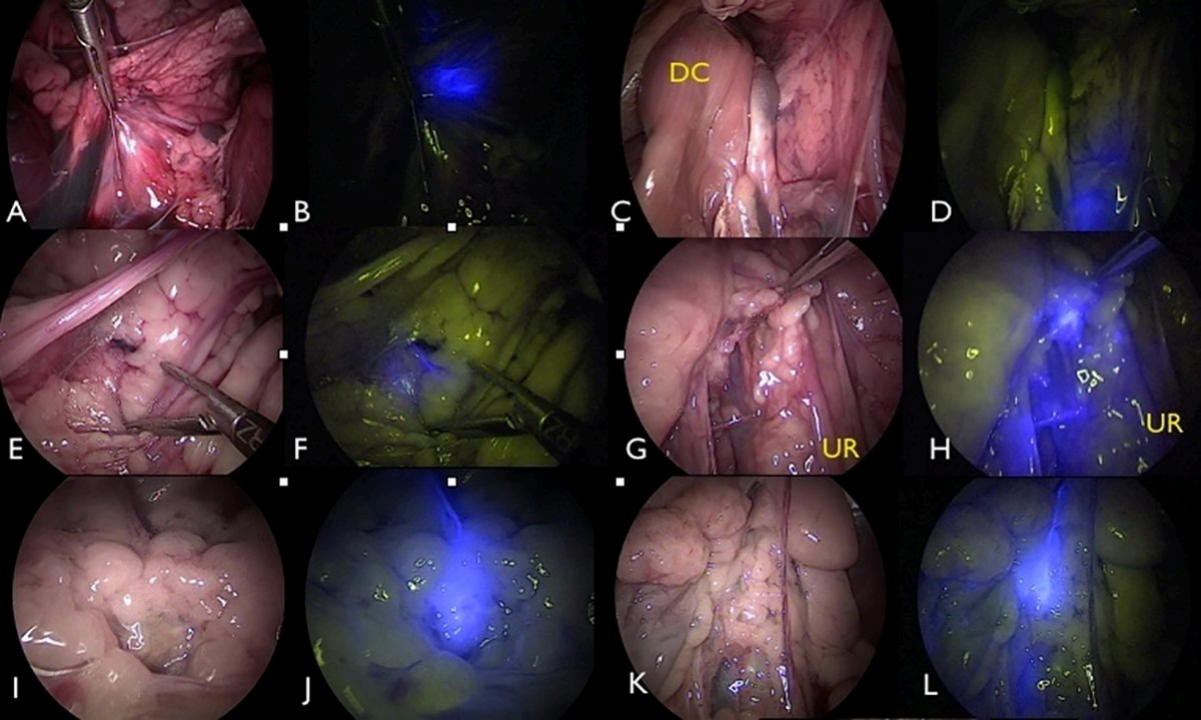
Laparoscopic fluorescence lymphography (LFL) in carcass 4 (C4) (A-D), C5 (E-H), and C6 (I-L). In A, B, E, F, I, and J, indocyanine green (ICG) was administered intradermally in the torso. In C, D, G, H, K, and L, ICG was administered via IPP. The blue stain shows the medial iliac lymph node (MILN) and/or efferent lymphatic pathways. In C4, the left MILN is shown without (A) and with (B) near-infrared fluorescence filtering. The right MILN is shown without (C) and with (D) near-infrared fluorescence filtering. In C5, IPP administration of ICG did not stain the left MILN but did stain the efferent lymphatic pathways (E and F). The right MILN is shown without (G) and with (H) near-infrared fluorescence filtering. In C6, ICG fluorescence enhanced MILN and lymphatic pathway identification (I-L). UR = ureter; DC = descending colon.

**Fig 5:**
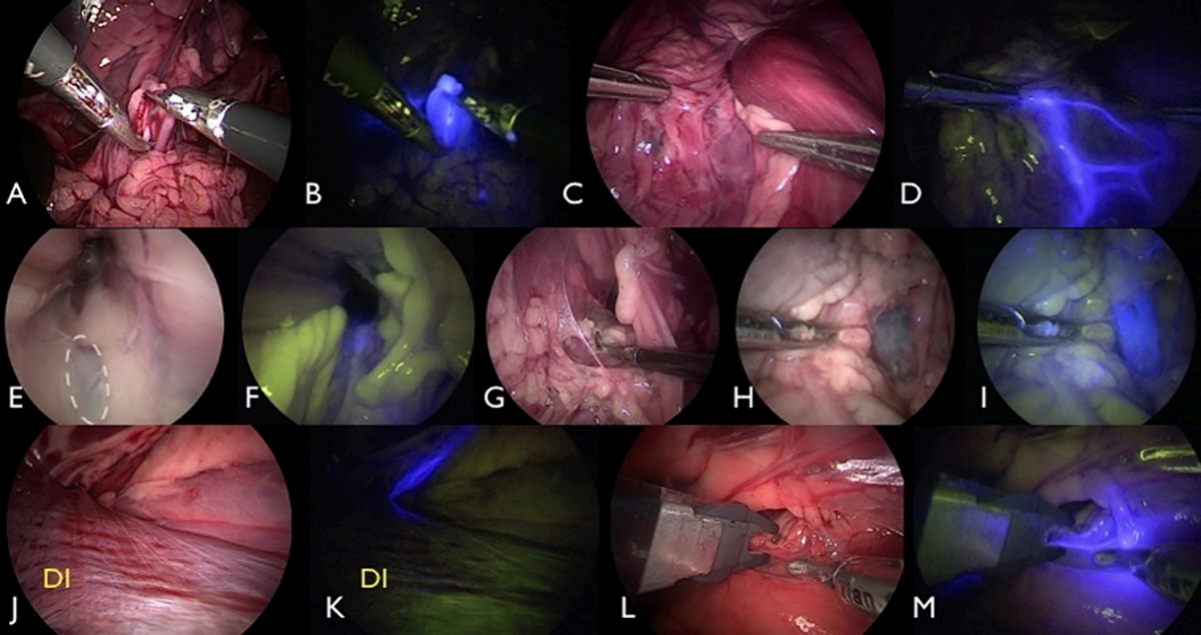
Left medial iliac lymph node (MILN) during laparoscopic lymphadenectomy without (A) and with (B) near-infrared fluorescence filtering in carcass 1 (C1). In C2, while the left MILN is not stained (C) but communication lymphatic vessels between the right and left MILN are stained (D). In C6, the right internal iliac lymph node (IILN_R_) was located by laparoscopic visualization (E, dotted circumference) but was not stained. In C5, the IILN_R_ was stained following intrapopliteal (IPP) administration of indocyanine green (ICG) (F), allowing its localization and lymphadenectomy by laparoscopy (G). In C6, the caudal vena cava (H) was stained (I) after IPP ICG administration. C2 was the only carcass in which the thoracic duct (J) was stained (K) by ICG. Laparoscopic fluorescence lymphography (LFL) using ICG allows easy identification of the thoracic duct (L and M) for occlusion by titanium clips. DI = diaphragm.

No statistically significant differences in ICG volume were observed between the ID and IPP administration routes (0.056±0.018 vs. 0.06±0.021 mg/kg; *p* = 0.73) for both intra-abdominal lymph vessel and MILN staining. In addition, both approaches had average time classifications of II and staining qualities of 2. MILNs were stained in all cases via IPP administration but in only three cases via ID (Figs 3–5). Thus, IPP administration was more effective than ID in staining MILNs. The IPP administration route required similar ICG volume (ID: 0.067±0.029 mg/kg; IPP: 0.06±0.022 mg/kg; *p* = 0.84) and time to stain the MILN (ID: III; IPP: II; *p* = 0.64) than those for ID. In contrast, IPP administration was technically more difficult than the corresponding ID and required surgical access for ICG administration.

In most cases, the intralymphonodal administration took less than 10 min (classification II) to stain the intracavitary afferent lymphatic pathway (4/5) and right MILN (3/5). ID administration allowed the visualization of luminescence in the afferent lymphatic vessels in less than 10 min (5/5) (Figs 3F and 4F). ID resulted in MILN staining in three cases (3/5), two of which received an ICG dose of 0.05 mg/kg (C4 and C5) and staining time of less than 10 min.

**Fig 6:**
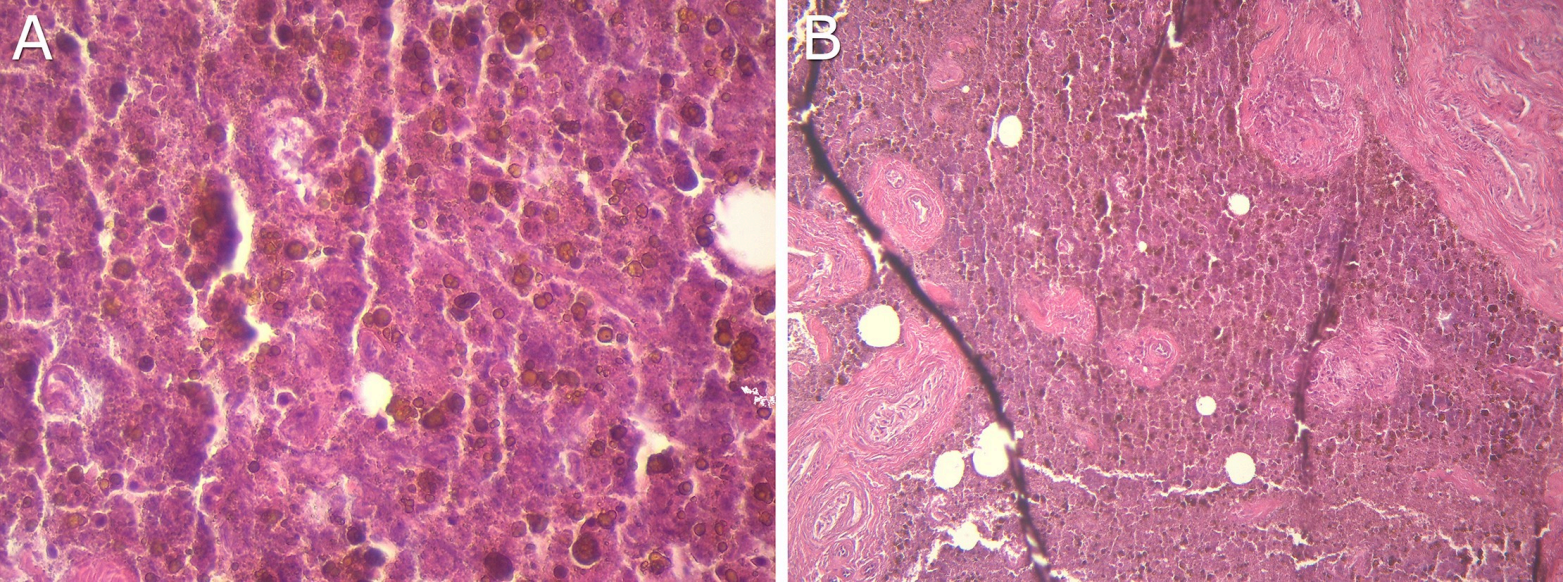
Histological sections. (a) Fragment of fibrovascular tissue artifacted with areas of amorphous granular background, with meatein crystals and in the absence of identifiable cellularity. (b) Trabecular adipose and fibrous tissue with an amorphous nodular aggregate with few identifiable hemophagocytic cells.

Regarding histological analysis, 62.5% of the samples collected from the carcasses were identified as lymphatic nodules, by both ID and IPP administration of ICG (Table 5). Two of the samples collected after IPP administration were classified as doubtful negatives (Fig 6). They were identified as fragments of fibrovascular tissue and as adipose and fibrous tissue, respectively, without identifiable cellularity.

**Table 5:** Histological analysis of samples collected from each carcass after ICG administration via ID and IPP.

Carcass (C#)	Anatomical region	ICG total volume (0.5 mg/mL)	Via	Side (left/right)	Histological analysis of lymphoid tissue	Macroscopic evaluation and comments
C1	Caudal iliac	5 mL	ID	Left	Negative	Tissue stained by ICG. Collected beneath the mesocolic LN. Macroscopic features of a remnant of another LN.
	Cranial iliac				Positive	Tissue stained by ICG. Brownish color.
	Cranial iliac	5 mL	IPP	Right	Positive	Tissue stained by ICG. Brownish color. Collected in fragments.
	Caudal iliac				Negative	Tissue stained by ICG. Brownish color. Collected in fragments. Dissected beneath the iliac vessels and not below the ureter.
C2	Medial iliac	8.8 mL	ID	Left	Negative	Not stained by ICG. Dark green color.
	Iliac	2.2 mL	IPP	Right	Positive	Tissue stained by ICG. Macroscopic features of fat tissue but potential micro LN in fat tissue.
	Pelvic				Positive	Tissue stained by ICG. Green color. Collected in fragments. Macroscopic features more like lymphatic aggregate than LN.
	Iliac				Negative	Tissue stained by ICG. Collected in the supine position.
C3	Medial iliac	1.65 mL	IPP	Right	Positive	Tissue stained by ICG. Green color. Collected in fragments. Macroscopic features of LN.
	Medial-caudal iliac				Positive	Tissue stained by ICG. Macroscopic features of LN.
C4	Medial iliac	4.2 mL	ID	Left	Positive	Tissue stained by ICG. Macroscopic features of LN.
	Medial-lateral iliac				Positive	Tissue stained by ICG.
	Femoral ring	1.4 mL	IPP	Right	Positive	Tissue stained by ICG. Collected in fragments. Macroscopic features of LN.
	Iliac				Negative	Tissue stained by ICG. Macroscopic features of LN.
C5	Medial iliac	4.95 mL	ID	Left	Positive	Tissue stained by ICG. Collected in fragments. Macroscopic features of LN.
	Medial iliac	4.95 mL	IPP	Right	Positive	Tissue stained by ICG. Brownish color. Collected in fragments. Macroscopic features of LN.
	Pelvic				Doubtful negative	Tissue stained by ICG. Brownish color. Macroscopic features of being part of another LN.
	Pelvic				Positive	Tissue stained by ICG. Greenish color. Collected in fragments.
	Pelvic				Doubtful negative	No ICG staining. Macroscopic features of micro LN.
C6	Medial iliac	3.15 mL	ID	Left	Positive	Tissue stained by ICG.
	Iliac				Negative	Tissue stained by ICG.
	Medial iliac	2.10 mL	IPP	Right	Positive	Tissue stained by ICG. Collected close to the right iliac/femoral vessels. Macroscopic features of LN.
	Femoral				Negative	Tissue fluorescent by ICG. Macroscopic features of lymphatic vessels.
	Pelvic				Positive	No ICG staining. Macroscopic features of LN.

LN: lymph node.

Regarding the durability of the ICG fluorescence, it was firstly administered via ID, followed by new administrations every 15 min if there was a lack of staining in the left MILN. Contralateral luminescence was still very evident at the end of the evaluation of the right MILN. Lymphadenectomy occurred after 66–113 min (> 88.2±17.0 min) of the initial ICG administration, with luminescence of the lymphatic vessels and MILNs persisting in all cases. In the only case (C2) in which the thoracic duct was stained, this structure was identified after 37 min of IPP injection (Fig 5J–5M).

## Discussion

The results of this study confirmed the feasibility of staining the lymphatic pathway and lymph nodes of the iliosacral lymphatic center (abdominal and pelvic) in thawed dog carcasses using direct near-infrared fluorescence with ICG by laparoscopy. These findings expand the possibilities of anatomical studies and training of video surgical techniques involving the canine lymphatic system without the use of in vivo models.

To our knowledge, no study has reported the staining time of MILNs in an ex vivo canine model by luminescence using ICG administered via ID or IPP. A previous study showed that the popliteal application of ICG in dogs required <10 min (time classification: II) to stain the thoracic duct in all animals where this pathway was effective (7/11). In most of these animals (4/7), the staining time was 2–5 min [3]. Enwiller et al. applied methylene blue via IPP to effectively stain the thoracic duct in 6/10 animals 5 minutes after administration [7]. During the SC administration of iopamidol around the anus, mediastinal lymph node staining was observed within the next 10 min [15]. In the case of transcutaneous detection of sentinel lymph nodes in the oral mucosa, the ipsilateral mandibular lymphocentrum was visually detected within 4–15 min [14].

Considering the initial staining times of the lymphatic pathways and MILNs, the results of this study verified that near-infrared fluorescence using ICG by laparoscopy also allows rapid intracavitary lymphography of thawed dog carcasses. This observation, together with the results of methylene blue lymphography and IPP administration of ICG in dogs [3, 7], led us to think that the staining time of MILNs by the two tested routes (ID and IPP) may be even lower in vivo models than in the carcasses. Factors present in the carcasses such as interference with the freezing and thawing process, dehydration, and possible obstruction of the lymphatic network by autolysis or the presence of ice crystals, will be non-existent in live animals.

Since this was an experimental study, it focused on establishing the luminescence times of each structure separately, which would not be of such significance in clinical practice since the goal would be to stain the sentinel lymph node(s) to facilitate lymphadenectomy and, thus, improve the quality of surgical dissection. Proper staining of sentinel lymph nodes would increase the likelihood of their removal without invading the capsule and reducing lesions in the adjacent structures. In addition, suitable staining would allow the identification of other lymph nodes associated with the target lymph center. Our findings regarding the duration of ICG staining in the lymph nodes and lymphatic pathway support the long-lasting characteristic of ICG fluorescence using laparoscopic near-infrared light in dogs.

In terms of the staining quality, grade 2 was achieved in all cases with stained structures (lymph vessels or lymph nodes), which is ideal for lymphadenectomy. The stain tended to expand into adjacent tissues, reducing the definition of the edges of the vessels and lymph nodes and sometimes reaching grade 3 on the evaluation scale. This pattern of dispersion through the lymphatic tissue may not be replicable in living patients, as this study used tissues that had lost some integrity. However, these findings suggest that, once the desired lymph node staining is achieved, lymphadenectomy should not be further delayed.

Regarding the ICG volume, most of the carcasses (C2–C4) required only one dose in the popliteal lymph node to stain the right internal iliac lymph node (IILN). Only C5 required three doses for visualization. One such lymph node was also identified in C6 but was not stained. The use of similar or larger ICG volumes via ID in the six analyzed carcasses did not allow the observation of other pelvic lymph nodes from the iliosacral lymphatic center (medial sacral, lateral sacral, and internal iliac lymph nodes) [18–20], demonstrating that the ID route was not adequate for visualizing these structures in dog carcasses. Based on previous findings on lymphatic pathways from the skin to the abdominal/pelvic lymphatics in dogs [17, 21], the administration of IGG via ID in the gluteal region near the base of the tail may allow identification of other lymph nodes in the iliosacral lymphatic center. Further studies on carcasses are necessary to verify this hypothesis.

The popliteal route drains to the MILN [17, 18]. However, this administration route may require surgical access [3] and is technically more difficult than ID administration. In addition, the torso region in dog carcasses also drains directly into the corresponding MILN [17, 21]. We confirmed this finding in all the carcasses in our study. This observation demonstrates the potential for the clinical application of this method in patients in which lymphadenectomy of this lymph node is indicated as an adjunct treatment or to determine the prognosis of certain types of neoplasms. The MILN receives efferent lymphatic vessels from the skin of the dorsal abdominal wall; the skin of the pelvis; the abdominal muscle; the muscle and bone of the posterior limb; the pelvic and lumbar muscles; and the colon, prostate, rectum, anus, vagina, testicles, ureter, lymph nodes (superficial and deep inguinal, left colic, sacral, and internal iliac), bladder, and urethra, among other tissues [18]. The MILN is a sentinel in the lumbar region and the last lymph node before draining into the venous circulation [21]. The MILN can also receive drainage from the breast chains [22]. Moreover, the MILN can metastasize from mammary neoplasms in female dogs without increasing in size [1].

Confirming observations from previous studies [22], we observed connections between the MILNs on each side in three of the six carcasses (C1, C2, and C4), indicating that lymphatic staining on one side could directly interfere with the other (Fig 5D). This fact could make it difficult to precisely distinguish the time required for luminescence in each MILN separately. This finding also indicates that, in dogs, the contralateral may also be affected in the presence of metastatic involvement of one of the MILNs.

The results of this study showed that the administration of ICG via ID in the torso allowed the rapid detection of fluorescence in the lymphatic pathway associated with the MILN in dog carcasses. There is currently a gap between the administration of ICG in dog carcasses and in live patients, in which the ideal ICG dose/kg and concentration are not yet well established for administration via ID. Suami et al. used 0.2 mL of 0.25% ICG in two dogs and reported no associated complications [9]. However, Souza et al. administered ICG via ID (1% ICG; 0.5 mL for female dogs <15 kg and 1 mL when >15 kg), to six animals, reporting significant local morbidity including skin necrosis and edema of the skin and erythema (consistent with type IV hypersensitivity), which required medical treatment [16]. As the present study aimed to compare the two administration routes (ID and IPP), the default dose was chosen for administration via IPP [3].

The carcass position and surgical port placement for the laparoscopic lymphadenectomies differed from those reported previously in a canine model [19, 20]. In the present study, triangulation of the trocars in the ventral midline (near the umbilical scar) together with the Trendelenburg position allowed us to complete the exploration of the abdominal and pelvic cavities in the region corresponding to the iliosacral lymphatic center, of which the MILN is part [18–20]. Since we believed that other nodes/micro lymph nodes could be found in addition to the MILN, the dorsal decubitus position was chosen to provide more working space for bilateral evaluations and extractions, since lateral access did not allow identification of the contralateral MILN [19]. The triangulation proposed for surgical ports differed from that proposed by Lim et al. [20], in which one of the trocars was placed in the inguinal region. The present study placed the three trocars cranially to the MILN to reduce instrumental mirror movements.

A previous study in a canine in vivo model showed that the popliteal route allowed ICG fluorescence of the thoracic duct in 7/11 animals with chylothorax [3]. In the present study, the fluorescence of the thoracic duct was also observed in one carcass (C2) (Fig 5J–5M). Several factors could interfere with ICG reaching the thoracic duct in the remaining thawed dog carcasses. However, the methodology followed for this study does not allow us to define the reason for this. As the aim of the study was to stain the iliosacral lymphatic center, the dose/concentration of ICG via IPP was not extended in search of the thoracic duct. However, staining of the thoracic duct in one thawed dog carcass also opens up a new possibility in terms of using ex vivo models for training of thoracic duct occlusion in dogs.

As for the complications observed, a section of the left ureter was accidentally made during the lymphadenectomy of the carcass C2. Analysis of the recorded laparoscopic videos revealed that the luminescence was dispersed as a result of the resection of the right MILN. It was also observed that the search for lymphatic tissue was carried out very close to the bladder neck and the collection was done with apparently not enough care, prioritizing a large resection of the stained adipose tissue. From this event, a careful blunt dissection of the stained structures was performed to subject them to histology, always verifying the position of the ureter and continuously changing the type of illumination of the surgical field (infrared light and normal light). Apart from that case, no other cases of lesions in the ureter or large vessels were found.

In carcass C6, intravenous fluorescence was observed in the pelvic and abdominal vessels, including the external iliac and cava vessels (Fig 5H and 5I). The cause could not be determined, but we believe that it was due to extravasation of ICG into the perinodal tissue or misapplication intravenous administration, since in this animal the lymph node was small and it had to be punctured at least three times.

This study has some limitations such as the restricted sample size, which will be taken into account for future studies in this field. Furthermore, the evaluation of ICG fluorescence in thawed dog carcasses has some constraints due to the nature of this sample. Several factors may interfere with adequate lymph node staining, including the quality of the thawing process, the permeability of the lymphatic pathway, and autolysis/decomposition of the carcass. Factors including massaging the site of ICG administration, the ICG volume, the anatomical characteristics of the lymphatic pathway and lymph nodes, and the amount of adipose tissue in the patient could also influence the timing of lymphatic fluorescence, correct identification of the lymph nodes, and perception of the exact staining time. Although near-infrared light can penetrate several millimeters [23], the amount of adipose tissue covering the lymphatic pathway could make it difficult to visualize its small branches, as seen in C5 and C6 in our study. We believe that luminescence was present in these two carcasses long before it was detected. However, only part of a lymph vessel could be seen after removing the adipose tissue covering it. Since one of the aims of this study was to evaluate lymphatic staining without the need for tissue dissection (mainly MILNs), we cannot exclude the possibility that some lymph nodes were stained but not identified. Despite the limitations associated with the model, our findings provide evidence of the potential application of these methods in anatomical, experimental, and clinical studies involving the video surgical evaluation of lymphatic pathways and lymph nodes through ICG luminescence in thawed dog carcasses. To our knowledge, this is the first study to demonstrate the effectiveness of laparoscopic evaluation of the iliosacral lymph center by ICG luminescence via ID or IPP administration.

## Conclusions

Both ID and IPP ICG administration allowed laparoscopic fluorescence lymphography of the iliosacral lymph center in thawed dog carcasses. However, the administration of ICG via IPP was more effective.

## Supporting information

S1 Table (XLSX)

S2 Table (XLSX)

## References

[1] Feranti JPS, Coradini GP, Linhares MT, Hartmann HF, Campos RV, Oliveira MT, et al Iliac lymphadenectomy following intrauterine mapping in a female dog with Breast Neoplasm. Acta Scient Vet. 2018 46(Suppl 1): 273. 1–4.

[2] Szczubial M, Lopuszynski W. Prognostic value of regional lymph node status in canine mammary carcinomas. Vet Comp Oncol. 2011; 9(4): 296–303. 10.1111/j.1476-5829.2011.00268.x 22077411

[3] Steffey MA, Mayhew PD. Use of direct near-infrared fluorescent lymphography for thoracoscopic thoracic duct identification in 15 dogs with chylothorax. Vet Surg. 2017; 00:1–10. 10.1111/vsu.12740 29105790

[4] Gelb HR, Freeman LJ, Rohleder JJ, Synder PW. Feasibility of contrast-enhanced ultrasound-guided biopsy of sentinel lymph nodes in dogs. Vet Radiol Ultrasound. 2010; 51(6): 628–633. 10.1111/j.1740-8261.2010.01712.x 21158235

[5] Justino RC, Cardoso GS, Trautwein LGC, Dessunti GT, Oliveira DV, Bernardes R, et al Uterine lymphangiography: comparison of two methods for locating the medial iliac lymph node. Pesq Vet Bras. 2014; 34(11): 1121–1126.

[6] Pinheiro LGP, Oliveira Filho RS, Vasques PHD, Filgueira PHO, Aragão DHP, Barbosa PME, et al Hemosiderin. A new marker for sentinel lymph node identification. Acta Cir Bras. 2009; 24(6): 432–436. 10.1590/s0102-86502009000600002 20011826

[7] Enwiller TM, Radlinsky MG, Mason DE, Roush JK. Popliteal and mesenteric lymph node injection with methylene blue for coloration of the thoracic duct in dogs. Vet Surg. 2003; 32: 359–364. 10.1053/jvet.2003.50044 12865998

[8] Souza FW, Brun MV, Nardi AB, Huppes RR, Quarterone C, Raposo TMM, et al Linfadenectomia laparoscópica em cadela com neoplasma mamario. Cienc Rural. 2013; 43(4): 750–753.

[9] Suami H, Scaglioni MF, Dixon KA, Tailor RC. Interaction between vascularized lymph node transfer and recipient lymphatics after lymph node dissection-a pilot study in a canine model. J Surg Res. 2016; 204(2): 418–427 10.1016/j.jss.2016.05.029 27565078 PMC5002893

[10] Suga K, Karino Y, Fujita T, Okada M, Kawakami Y, Ueda K, et al Cutaneous drainage lymphatic map with interstitial multidetector-row computed tomographic lymphography using iopamidol: preliminary results. Lymphology. 2007; 40(2): 63–73. 17853616

[11] Iida G, Asano K, Seki M, Ishigaki K, Teshima K, Yoshida O, et al Intraoperative identification of canine hepatocellular carcinoma with indocyanine green fluorescent imaging. J Small Anim Pract. 2013; 54(11): 594–600. 10.1111/jsap.12148 24580016

[12] Araki J, Nishizawa Y, Nakamura T, Sato T, Naito M, Fujii S, et al The development of a canine anorectal autotransplantation model based on blood supply: a preliminary case report. PLoS One. 2012; 7(9): e44310 10.1371/journal.pone.0044310 22970198 PMC3435401

[13] Hassan M, Kerdok A, Engel A, Gersch K, Smith JM. Near infrared fluorescence imaging with ICG in TECAB surgery using the da Vinci Si surgical system in a canine model. J Card Surg. 2012; 27(2):158–162. 10.1111/j.1540-8191.2011.01411.x 22372818

[14] Townsend KL, Milovancev M, Bracha S. Feasibility of near-infrared fluorescence imaging for sentinel lymph node evaluation of the oral cavity in healthy dogs. Am J Vet Res. 2018; 79(9):995–1000. 10.2460/ajvr.79.9.995 30153060

[15] Iwanaga T, Tokunaga S, Momoi Y. Thoracic duct lymphography by subcutaneous contrast agent injection in a dog with chylothorax. Open Vet J. 2016; 6(3): 238–241. 10.4314/ovj.v6i3.13 27995081 PMC5155138

[16] Souza FW, Brun MV, Feranti JPS, Oliveira MT, Copat B, Baumer S, et al Laparoscopic inguinoiliac lymphadenectomy following staining using different lymphatic markers in healthy dogs. Cienc Rural. 2016; 46(9): 1629–1634.

[17] Suami H, Yamashita S, Soto-Miranda MA, Chang DA. Lymphatic territories (lymphosomes) in a canine: an animal model for investigation of postoperative lymphatic alterations. PLoS One. 2013; 8(7): e69222 10.1371/journal.pone.0069222 23894435 PMC3722290

[18] Bezuidenhout AJ. The Lymphatic System In: Evans HE, editor. Miller’s Anatomy of the Dog. 3rd ed Philadelphia: W.B. Saunders company; 1993 pp. 717–757.

[19] Steffey MA, Daniel L, Mayhew PD, Affolter VK, Soares JH, Fuller MC. Laparoscopic Extirpation of the Medial Iliac Lymph Nodes in Normal Dogs. Vet Surg. 2015; 44(suppl 1): 59–65. 10.1111/j.1532-950X.2014.12207.x 24899462

[20] Lim H, Kim J, Li L, Lee A, Jeong J, Ko J, et al Bilateral medial iliac lymph node excision by a ventral laparoscopic approach: technique description. J Vet Med Sci. 2017; 79(9):1603–1610. 28781294 10.1292/jvms.16-0627 PMC5627336

[21] Suami H, O’Neill JK, Pan W, Taylor GI. Perforating lymph vessels in the canine torso: direct lymph pathway from skin to the deep lymphatics. Plast Reconstr Surg. 2008; 121(1): 31–36. 10.1097/01.prs.0000293753.93274.21 18176203

[22] Reese S, Budras KD, Mulling Chr, Bragulla H, Konig HE. Integumentum commune In: König H, Liebich HG, editors. Anatomia dos animais domésticos. 6th ed São Paulo: ArtMed; 2016 pp. 615–66.

[23] Gioux S, Choi HS, Frangoni JV. Image-Guided Surgery using Invisible Near-Infrared Light: Fundamentals of Clinical Translation. Mol Imaging. 2010; 9(5): 237–255. 20868625 PMC3105445

